# Resolving the difference between left-sided and right-sided colorectal cancer by single-cell sequencing

**DOI:** 10.1172/jci.insight.152616

**Published:** 2022-01-11

**Authors:** Wei Guo, Cuiyu Zhang, Xia Wang, Dandan Dou, Dawei Chen, Jingxin Li

**Affiliations:** 1 Department of Colorectal Surgery, Shandong University Qilu Hospital, Jinan, Shandong, China.; 2 Department of Physiology, School of Basic Medical Sciences, Cheeloo College of Medicine, Shandong University, Jinan, Shandong, China.; 3 Laboratory of Medical Chemistry, Interdisciplinary Cluster for Applied Genoproteomics (GIGA) Stem Cells, University of Liege, CHU de Liège, Sart-Tilman, Liege, Belgium.

**Keywords:** Gastroenterology, Cancer

## Abstract

Colorectal cancers (CRCs) exhibit differences in incidence, pathogenesis, molecular pathways, and outcome depending on the location of the tumor. The transcriptomes of 27,927 single human CRC cells from 3 left-sided and 3 right-sided CRC patients were profiled by single-cell RNA-Seq (scRNA-Seq). Right-sided CRC harbors a significant proportion of exhausted CD8^+^ T cells of a highly migratory nature. One cluster of cells from left-sided CRC exhibiting states preceding exhaustion and a high ratio of preexhausted/exhausted T cells were favorable prognostic markers. Notably, we identified a potentially novel RBP4^+^NTS^+^ subpopulation of cancer cells that exclusively expands in left-sided CRC. Tregs from left-sided CRC showed higher levels of immunotherapy-related genes than those from right-sided CRC, indicating that left-sided CRC may have increased responsiveness to immunotherapy. Antibody-dependent cellular phagocytosis (ADCP) and antibody-dependent cellular cytotoxicity (ADCC) induced by M2-like macrophages were more pronounced in left-sided CRC and correlated with a good prognosis in CRC.

## Graphical abstract

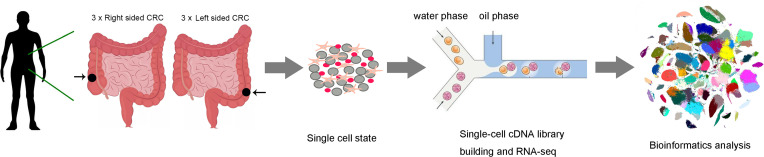

## Introduction

Often grouped as one disease, right-sided colorectal cancer (CRC; originating from cecum, ascending colon, hepatic flexure) and left-sided CRC (originating from splenic flexure, descending colon, sigmoid colon) represent clinically distinct entities with significant differences in their prognosis and treatment outcomes (1, 2). Right-sided CRC has a worse prognosis than left-sided CRC (3–5). Extensive sequencing analysis described a characteristic branching pattern of cancer evolution supporting that tumor biology is characterized simultaneously by intratumor heterogeneity and the preservation of ancestral aberrations within the primary tumor and corresponding metastatic sites (6, 7). However, the full spectrum of distinct cell types and their molecular characteristics remain to be well defined in left-sided and right-sided malignant colorectal lesions, which hampers our ability to investigate their differences in CRC pathogenesis.

Advances in single-cell RNA-Seq (scRNA-Seq) have revolutionized our ability to characterize the transcriptional state of thousands of individual cells in an in-depth manner. Here, we performed a scRNA-Seq survey of 27,927 cells from 6 samples obtained during curative surgery for 3 left-sided CRCs and 3 right-sided CRCs, and we constructed a single-cell transcriptome atlas for malignant colorectal lesions. We hypothesized that the reasons for the better prognosis of left-sided CRC compared with right-sided CRC might be the number and functional status of different immune cell subpopulations in the tumor microenvironment (TME) in CRC, as well as the level of different signaling pathways of cancer cells themselves and the interaction between cancer cells and TME cells. We used the atlas to construct a network for dissecting the cellular and molecular characteristics of left-sided and right-sided CRC.

## Results

### Single-cell atlas of CRC from left-sided and right-sided CRC patients.

**Figure 1:**
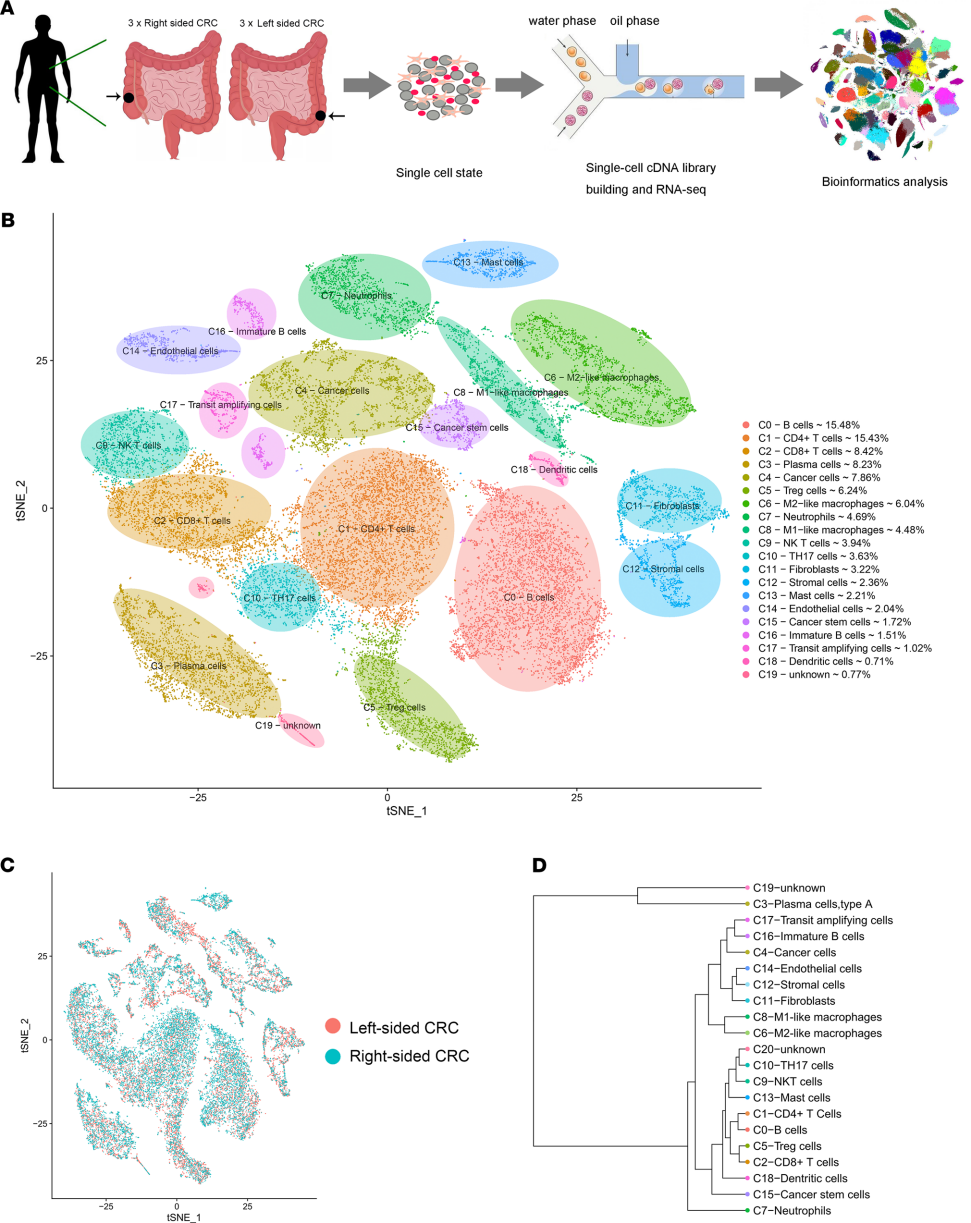
Single-cell atlas of colorectal cancer from left-sided and right-sided CRC patients. (**A**) Schematic diagram highlighting the experimental workflow for the whole study. (**B**) The t-SNE plot of 27,927 high-quality cells to visualize cell-type clusters based on the expression of known marker genes. (**C**) The t-SNE plot of all types of cells from left-sided CRC samples and right-sided CRC samples. (**D**) Unsupervised hierarchical clustering of average gene signatures showing relatedness of cell clusters (correlation distance metric, average linkage).

We generated 27,927 high-quality single-cell transcriptomes from 6 samples obtained during curative surgery for 3 left-sided and 3 right-sided CRCs (Figure 1A and Supplemental Table 1; supplemental material available online with this article; https://doi.org/10.1172/jci.insight.152616DS1). The quality control (QC) criteria are described in the Materials and Methods.

**Figure 2:**
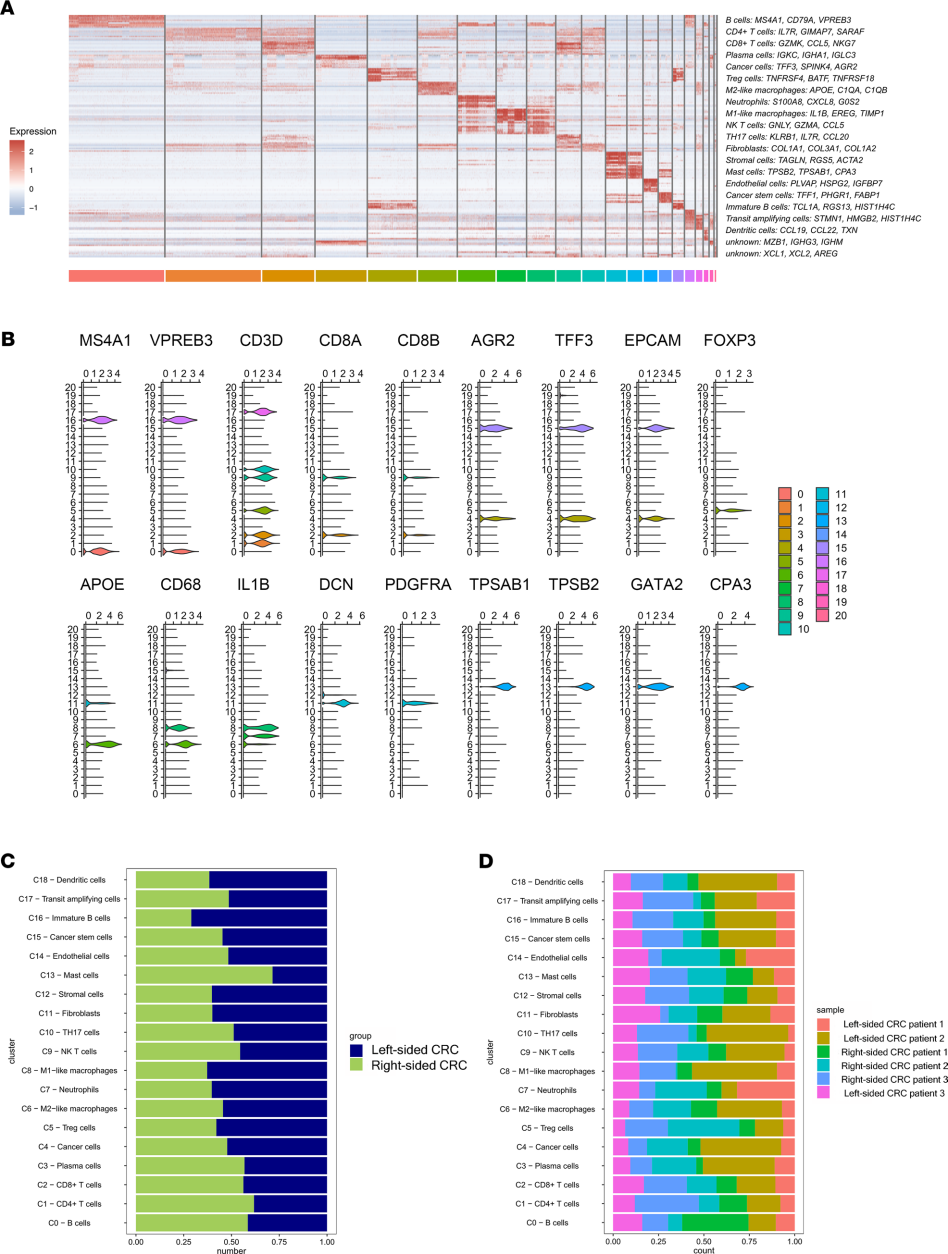
Single-cell atlas of colorectal cancer from left-sided and right-sided CRC patients. (**A**) Heatmap of differentially expressed genes. For each cluster the top 3 genes and their relative expression levels in all CRC cells are shown. (**B**) Violin plots display the distribution of expression of known marker genes across diverse cell types among CRC. (**C** and **D**) For 20 subgroups identified in this profile (left to right): the fraction of cells that originated from left-sided and right-sided CRC samples, and the fraction of cells that originated from each of the 6 patients.

After performing unsupervised clustering and t-distributed stochastic neighbor (t-SNE) plot analysis (Figure 1B), cluster identities were determined according to the expression of established markers (Supplemental Figure 1). We also noticed that several cell clusters were enriched in both left-sided and right-sided CRC (Figure 1C). A total of 13,488 single cells originated from left-sided CRC, while 14,439 originated from right-sided CRC (Figure 1C). We utilized the differentially expressed gene (DEG) signatures and attributed clusters to their putative identities and hierarchical similarities (Figure 1D and Supplemental Figure 1). Figure 2A shows selected DEGs in the form of a heatmap (Figure 2A) and feature plots (Figure 2B). Mast cells from right-sided CRC accounted for 71.5% of all cluster 13 cells, while left-sided CRC only accounted for 28.5% (Figure 2C). The proportion of each sample in these clusters was showed in Figure 2D.

These cells were classified into 19 main cell lineages (the last 2 unknown clusters were incorporated into cluster 19). In addition to cancer cells, we identified 13 immune cell lineages, including B cells, CD4^+^ T cells, CD8^+^ T cells, Tregs, macrophages, neutrophils, NK T cells, DCs, IL-17–producing Th17 cells, and mast cells, along with 4 nonimmune cell lineages (CD45^–^), including stromal cells, fibroblasts, endothelial cells, and transit amplifying cells (Figure 1B).

### Cell-specific expression changes in left-sided and right-sided CRC.

**Figure 3:**
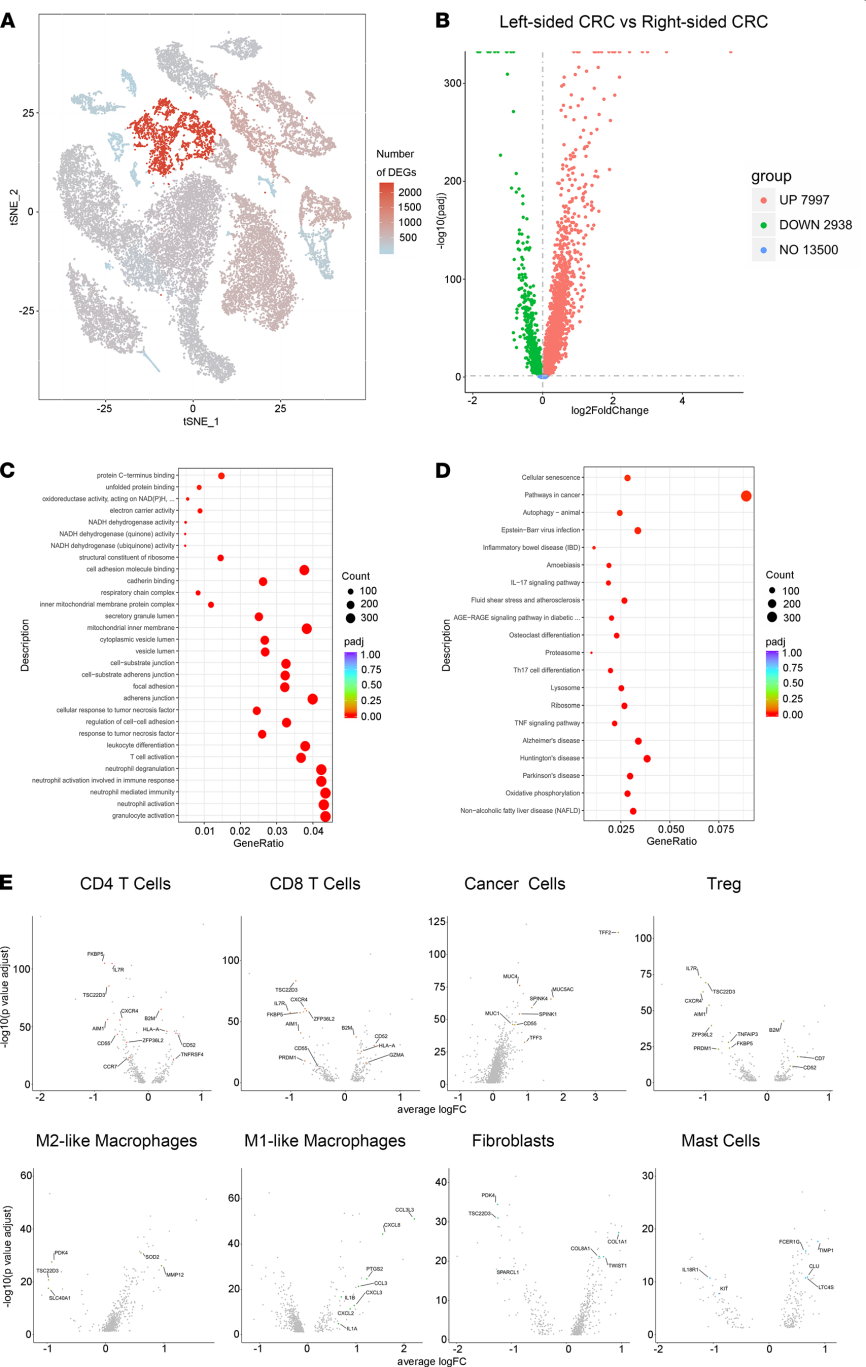
Cell-specific expression changes in left-sided and right-sided CRC. (**A**) Number of DEGs between left-sided and right-sided CRC cells within each cluster projected onto the t-SNE map. DEG, |log fold change| > 0.5; adjusted *P* < 0.05 was derived by a Wilcoxon rank-sum test. (**B**) A volcano plot of DEGs that are upregulated (red) or downregulated (green) between left-sided and right-sided CRC. (**C** and **D**) A cluster profiler identified the enriched gene ontology and Kyoto Encyclopedia of Genes and Genomes (KEGG) processes of DEG. (**E**) Unique changes in specific cell subsets between left-sided and right-sided CRC within CD4^+^ T cells, CD8^+^ T cells, cancer cells, Tregs, M1- and M2-like macrophages, fibroblasts, and mast cells compartments. Two-tailed paired Student’s *t* test was used to determine significance.

Projecting the number of DEGs onto the t-SNE plot revealed that cluster 4 cancer cells exhibited the most prominent transcriptomic changes compared with other cell types in the TME of CRC (Figure 3A), and this indicated that tumor cell population harbored the most essential transcriptomic differences between left-sided and right-sided CRC.

To understand the biological significance of transcriptional changes between left-sided and right-sided CRC, we performed pathway enrichment analysis with DEGs obtained via unsupervised clustering analysis (Figure 3B). Kyoto Encyclopedia of Genes and Genomes (KEGG) enrichment analysis of the DEGs indicated that a handful of genes was associated with neutrophil function (e.g., neutrophil-mediated immunity, neutrophil activation, neutrophil activation involved in immune activation, neutrophil degranulation, and granulocyte activation), T cell activation, cell adhesion molecule binding, and adherens junctions (Figure 3C). Gene ontology (GO) enrichment analysis of the DEGs indicated that a handful of genes was associated with pathways in cancer (Figure 3D).

To identify changes in expression associated with the functional state of different cell types, we distinguished DEGs across cell subsets in CD4^+^ T cells, CD8^+^ T cells, Tregs, M1-like macrophages, M2-like macrophages, and fibroblasts in the form of volcano plots (Figure 3E).

### Naive CD4^+^ T cells are predominant in right-sided CRC.

**Figure 4:**
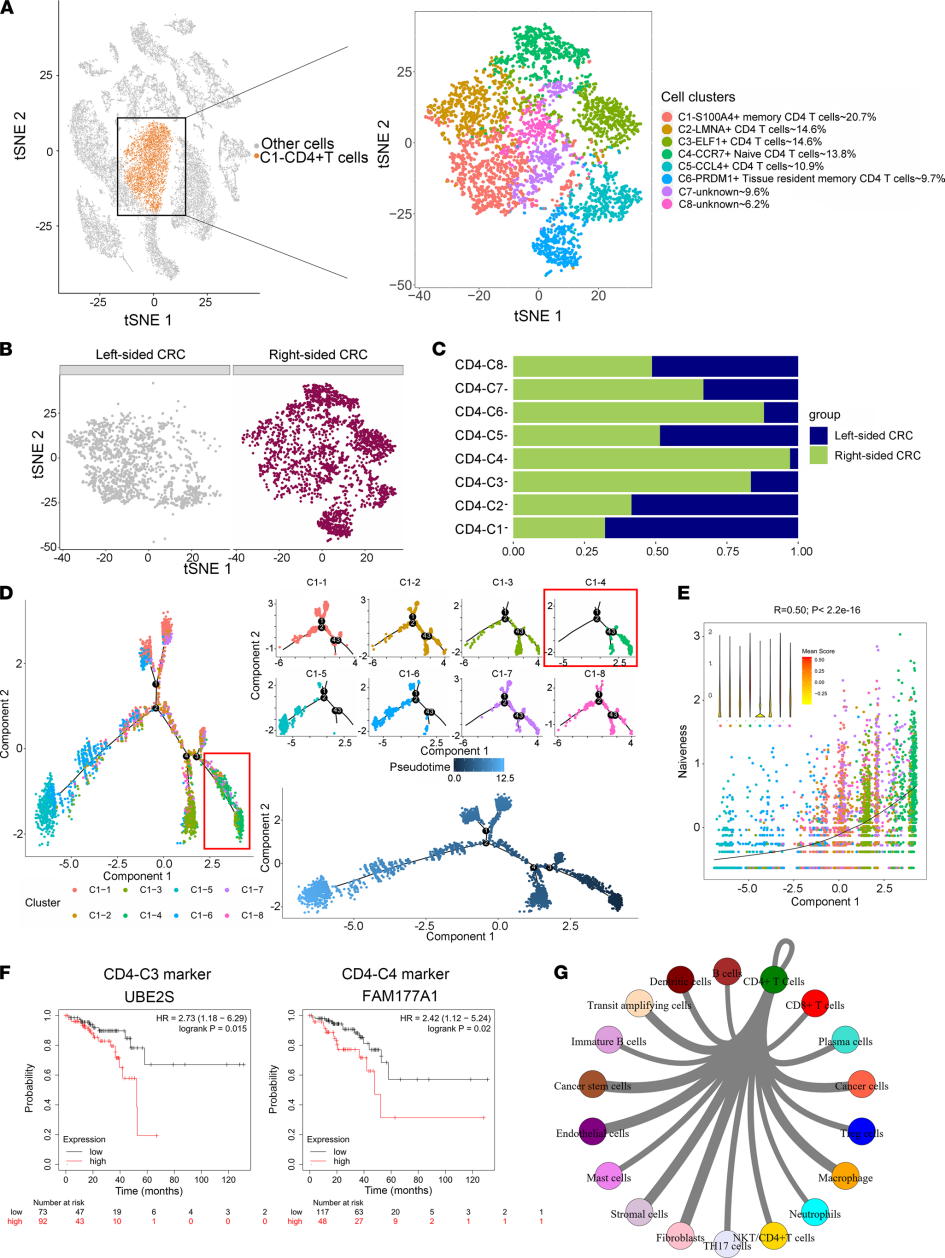
Naive CD4^+^ T cells are predominant in right-sided CRC. (**A**) The t-SNE plot that showed the distribution of CD4^+^ T cell lineages (orange, *n* = 4310 cells) within the atlas. CD4^+^ T cell populations were reclustered into 8 subclusters (color coding). (**B**) Annotation by left-sided and right-sided CRC cells. (**C**) The fraction of cells that originated from left-sided and right-sided CRC samples for 8 subgroups identified in this profile. (**D**) Differentiation trajectory of CD4^+^ T cells in CRC, with each color coded for pseudotime and clusters. (**E**) Monocle components were correlated with functional features of CD4^+^ T cells (the 4310 cells as in **A**), including scores of naiveness calculated by the mean expression of gene sets related to this T cell status (see Methods). (**F**) Kaplan-Meier survival curves of OS based on UBE2S and FAM177A1 expression using the online bioinformatics tool Kaplan-Meier Plotter. (**G**) The similarity network between CD4^+^ T cell and diverse cell types in our data set. The thickness of edges in the network was denoted as the Pearson correlation coefficient between the centroids of any pair of cell types.

CD4^+^ T cells were then clustered into 8 subgroups (Figure 4, A and B). We examined the specific genes expressed by each CD4 subgroup to identify their functional status. CD4 cluster 4 (CD4-C4) was enriched for CCR7, a specific marker for naive CD4^+^ T cells (Figure 4C). The trajectory was visualized as a t-SNE plot. We noticed that CD4-C4 was present at the start of the differentiation trajectory (Figure 4D). To better understand the trajectories, we defined scores based on previously defined gene signatures (8), and we found that component 1 (abscissa axis in differentiation trajectory) was highly associated with T cell naiveness, and CD4-C4 exhibited the highest naiveness score (Figure 4E). CD4-C6, representing approximately 9.7% of all CD4^+^ T cells, was characterized by high expression of PRDM1, suggesting that CD4-C6 was most likely tissue resident memory (Trm) CD4^+^ T cells (Supplemental Figure 2).

It was reported that tumor-infiltrating Tregs develop not only from recruited Tregs but also from naive T cells in situ in human breast cancer. The abundance of naive CD4^+^ T cells and Tregs is closely correlated, and both indicate poor prognosis for breast cancer patients (9). Our data show that the number of naive CD4^+^ T cells from right-sided CRC was 9-fold higher than that of data from left-sided CRC. Our CRC data were in line with the above observations from breast cancer. Kaplan-Meier survival curves of overall survival (OS) based on UBE2S (CD4-C3 marker) and FAM177A1 (CD4-C4 marker) expression indicated a poor prognosis (Figure 4F). The similarity network between CD4^+^ T cells and other cell types in our data set is shown in Figure 4G.

### Right-sided CRC occupies a large proportion of highly migratory exhausted CD8^+^ T cells.

**Figure 5:**
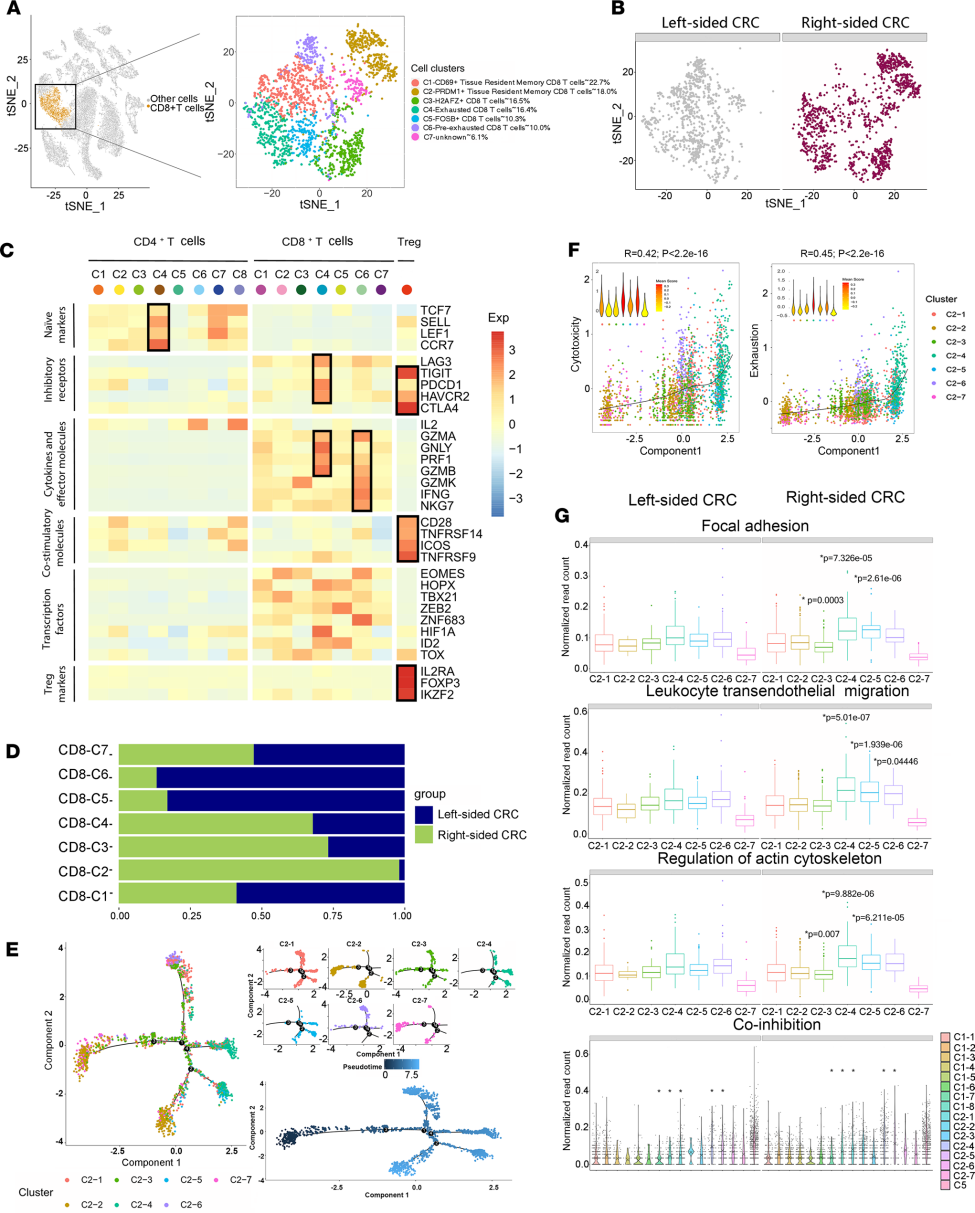
Right-sided CRC occupies a large proportion of highly migratory exhausted CD8^+^ T cells. (**A**) The t-SNE plot that showed the distribution of CD8^+^ T cell lineages (orange, *n* = 2351 cells) within the atlas. CD8^+^ T cell populations were reclustered into 7 subclusters (color coding). (**B**) Annotation by left-sided and right-sided CRC cells. (**C**) *Z* score normalized mean expression of selected T cell function–associated genes in each cell cluster. Black boxes highlight the prominent patterns defining known T cell subtypes. (**D**) The fraction of cells that originated from left-sided and right-sided CRC samples for 7 subgroups identified in this profile. (**E**) Differentiation trajectory of CD8^+^ T cells in CRC, with each color coded for pseudotime and clusters. (**F**) Monocle components were correlated with functional features of CD8^+^ T cells (the 2351 cells as in **A**), including scores of exhaustion and cytotoxicity calculated by the mean expression of gene sets related to T cell status. (**G**) Box plots of the expressions of Kyoto Encyclopedia of Genes and Genomes (KEGG) pathways and coinhibition program of all CD4^+^ and CD8^+^ T cell clusters between left-sided and right-sided CRC. *P < 0.05; Two-tailed paired Student’s *t* test was used to determine significance.

We applied unsupervised clustering based on t-SNE and identified 7 CD8^+^ T cell clusters (Figure 5, A and B). Next, we examined the expression of T cell–associated signature genes and known functional markers to define their identities.

CD4-C4 was characterized by high expression of genes associated with naiveness, including CCR7, SELL, LEF1, and TCF7 (Figure 5C). We found that some clusters exhibited distinct expression patterns among all CD8^+^ T cells in our data set. Cluster 4 was enriched for the effector T cell marker GNLY and the cytotoxicity-associated gene GZMB. Cluster 4 was characterized by high expression of genes associated with cytotoxicity, including GNLY, PRF1, GZMA, and GZMB, also showing high expression of T cell exhaustion markers such as PDCD1, LAG3, and HAVCR2. These data suggest that cluster 4 was exhausted CD8^+^ T cells (Figure 5C). Interestingly, we observed that some cluster of CD8^+^ T cells exhibited states preceding exhaustion. Cluster 6 represented ~10% of all CD8^+^ T cells, and its specific markers included genes associated with cytotoxicity, such as GZMH and GZMK, and chemokines, such as CCL3L3 and CCL4L2. Cluster 6 was characterized by high expression of genes associated with cytotoxicity, including PRF1, GZMA, GZMB, GZMK, IFNG, and NKG7 but had low expression of T cell exhaustion markers such as PDCD1, LAG3, TIGIT, CTLA4, and HAVCR2 (Figure 5C).

Right-sided CRC–derived exhausted CD8^+^ T cells accounted for as many as 67.9% of all CD8-C4 cells, while left-sided CRC–originated preexhausted effector CD8^+^ T cells accounted for 86.8% of all CD8-C6 cells. The ratio of preexhausted to exhausted T cells in left-sided CRC was 13.8-fold higher than right-sided CRC (Figure 5D). It has been reported that a high ratio of preexhausted to exhausted T cells is associated with a better prognosis than a low ratio in lung adenocarcinoma (8).

CD8-C1 and CD8-C2 were characterized by high expression of PRDM1 and CD69, suggesting these 2 subgroups were most likely Trm CD8^+^ T cells (Figure 5D and Supplemental Figure 2).

We applied an unsupervised inference method Monocle to construct the potential developmental trajectories of 7 CD8 clusters. CD8-C4 exhaustion cluster and CD8-C6 preexhaustion cluster positioned at different ends of the developmental trajectory. A part of the CD8-C2 PRDM1^+^ Trm CD8^+^ T cell cluster was positioned at the start of the developmental trajectory (Figure 5E). To better understand the trajectories, we defined cytotoxicity scores based on previously defined gene signatures (10, 11) and T cell exhaustion scores based on the average expression of 90 genes highly expressed in tumor-infiltrating exhausted CD8^+^ T cells (8). We analyzed the Monocle trajectory in the context of these functional scores, and we found that component 1 was highly associated with T cell exhaustion and cytotoxicity (Figure 5F).

It was reported that the presence of highly migratory preexhausted effector T cells in tumors provides a plausible explanation for the positive response to immunotherapies for non–small cell lung cancer (NSCLC) patients (8). It was also reported that increased pathogen-specific T cell numbers together with altered migratory patterns can greatly improve immune efficacy (12). Our data show that CD8-C4–exhausted CD8^+^ T cells from right-sided CRC showed higher levels of components of the focal adhesion, leukocyte transendothelial migration, and regulation of the actin cytoskeleton pathways than those from left-sided CRC (Figure 5G). T cell exhaustion is one of the mechanisms by which cancer cells evade the immune system. We concluded that exhausted CD8^+^ T cells from right-sided CRC were more prone to migrate to organs outside the TME, including lymph nodes, liver, and lung, leading to a higher tendency of metastasis compared with left-sided CRC.

The T cell signatures for the coinhibition program (CTLA4, PDCD1, TIGIT, HAVCR2, LAG3, BTLA, PDPN, CD160, GP49A, LILRB4, CD274, CD200, CD244, PILRA, SIRPB1, LAIR1, CEACAM1, KLRA7, KLRA3. KLRA9, PTGER4, KLRD1, KLRC1, and PROCR) were derived from known markers (8). We observed that most subsets of CD8^+^ T cells induced coinhibitory programs, and CD8-C1 CD69^+^ Trm cells, CD8-C4 exhausted CD8^+^ T cells, and CD8-C5 FOSB^+^ CD8^+^ T cells from right-sided CRC induced stronger coinhibitory programs compared with those from left-sided CRC (Figure 5G).

### Tregs from left-sided CRC exhibit higher level of immunotherapy-related genes.

Tregs suppress the antitumor function of effector T cells and NK cells by secreting soluble immunosuppressive factors and expressing inhibitory receptors (13, 14). A high proportion of Tregs in tumor-infiltrating T cells is associated with a poor prognosis in various types of human cancers (15). The prevailing idea is that Tregs are recruited from preexisting circulating Tregs by chemokines or chemokine ligands expressed by tumor cells, stroma, or tumor-associated macrophages (TAMs) (16, 17). An alternative possibility is that naive or conventional T cells might be recruited to the tumor and differentiate into Tregs in situ within the immunosuppressive tumor environment (13, 18, 19).

**Figure 6:**
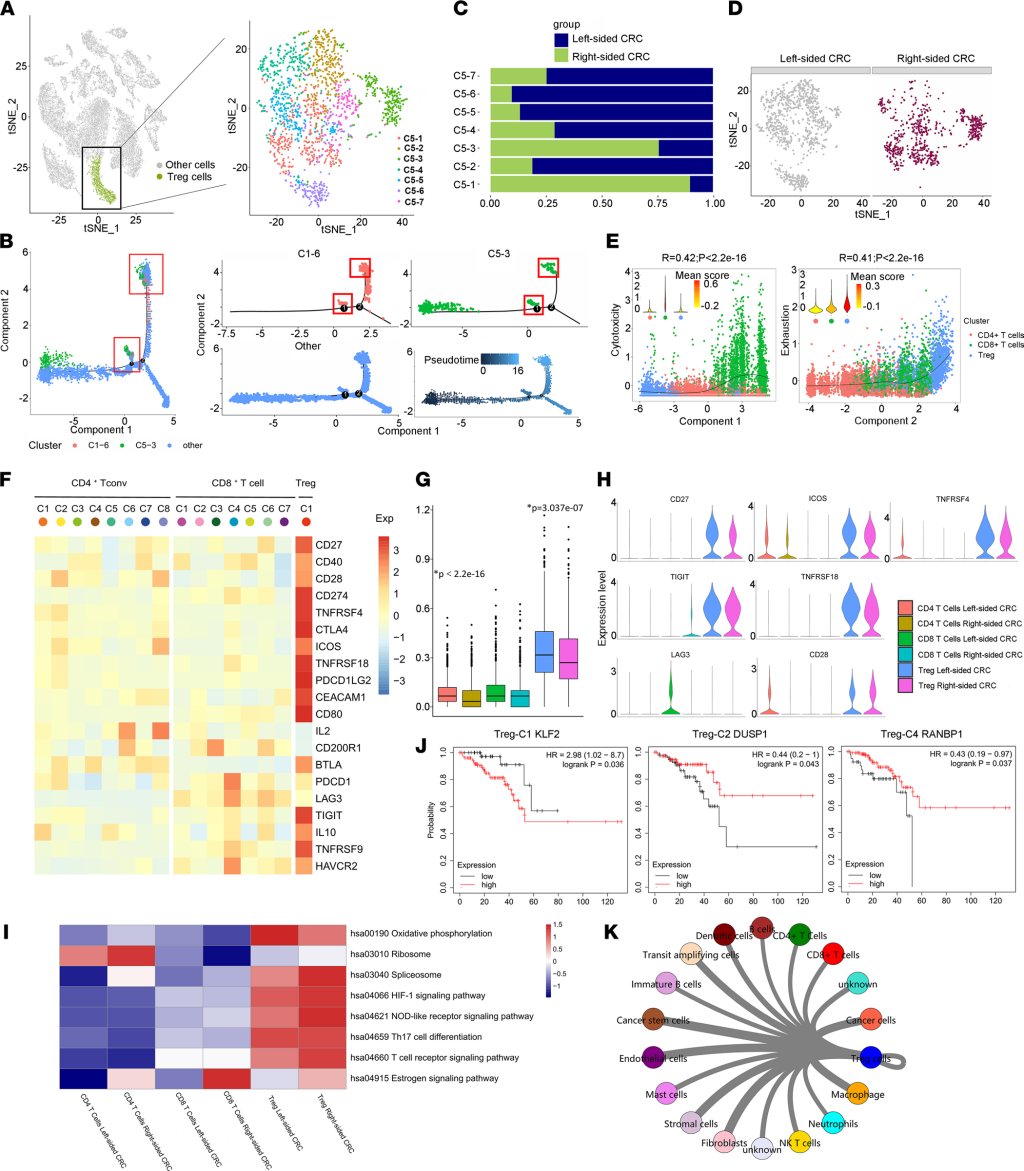
Tregs from left-sided CRC exhibit higher level of immunotherapy-related genes. (**A**) The t-SNE plot that showed the distribution of Treg lineages (green, *n* = 1742 cells) within the atlas. Treg cell populations were reclustered into 7 subclusters (color coding). (**B**) Differentiation trajectory of CD4^+^ T cells and Tregs in CRC, with each color coded for CD4-C6 (tissue resident memory CD4^+^ T cells), Treg-C3 and pseudotime. (**C**) The fraction of cells that originated from left-sided and right-sided CRC samples for 7 subgroups identified in this profile. (**D**) Annotation by left-sided and right-sided CRC cells. (**E**) Monocle components were correlated with functional features of Tregs (the 1742 cells as in **A**), including scores of exhaustion and cytotoxicity calculated by the mean expression of gene sets related to the T cell status. (**F**) Heatmap of the expression patterns of genes currently targeted by immunotherapies. (**G**) Box plots of mean expressions of genes currently targeted by immunotherapies of all CD4^+^, CD8^+^ T cell, and Treg clusters between left-sided and right-sided CRC. (**H**) Violin plots display the distribution of expression of CD27, ICOS, TNFRSF4, TIGIT, TNFRSF18, LAG3, and CD28 across CD4^+^ T cell, CD8^+^ T cell, and Tregs among CRC. (**I**) Kaplan-Meier survival curves of OS based on KLF2, DUSP1, and RANBP1 expression using the online bioinformatics tool Kaplan-Meier Plotter. (**J**) Differences in 8 hallmark pathway activities scored with GSVA software. Shown are *t* values calculated by a linear model. (**K**) The similarity network between Treg and diverse cell types in our data set. The thickness of edges in the network was denoted as the Pearson correlation coefficient between the centroids of any pair of cell types. *P < 0.05. Two-tailed paired Student’s *t* test was used to determine significance.

Treg-C1 represented ~20% of all Tregs, and the specific markers included genes associated with chemokines or chemokine ligands, such as CCL13, CCR7, and CXCR4 (Figure 6A and Supplemental Figure 2). This indicated that Treg-C1 cells were highly likely recruited from preexisting circulating Tregs by tumor cells, stroma, or TAMs from the TME. Treg-C3 was enriched for PRDM1, a specific marker for Trm T cells. We found that Treg-C3 cells shared similar marker gene signatures with CD4-C6 cells (Supplemental Figure 2) and Trm CD4^+^ T cells, leading us to wonder whether this subgroup of intratumoral Tregs mainly develops from PRDM1^+^ Trm CD4^+^ T cells. We analyzed the Monocle trajectory for 8 clusters of CD4^+^ T cells and 7 clusters of Tregs, and the results showed that some Treg-C3 cells shared the same position as CD4-C6 cells. Our data suggest that Trm CD4^+^ T cells might differentiate into Treg-C3 cells in situ within the immunosuppressive tumor environment (Figure 6B).

The number of Treg-C1 cells from right-sided CRC was 9-fold higher than that of those from left-sided CRC. Similarly, the number of Treg-C3 cells from right-sided CRC was 3-fold higher than that of those from left-sided CRC (Figure 6, C and D).

We analyzed the Monocle trajectory for CD4^+^ T cells, CD8^+^ T cells, and Tregs in the context of functional scores, and we found that component 2 was highly associated with T cell exhaustion, whereas component 1 was positively associated with cytotoxicity (Figure 6E). CD8^+^ T cells showed the highest cytotoxicity level, and Tregs showed the highest T cell exhaustion level.

PDCD1 exhibited high expression in exhausted CD8^+^ T cells (CD8-C4), and suppressive tumor Tregs highly expressed CTLA4. This is in line with recent observations that anti-CTLA4 and anti-PD1 therapies target distinct tumor-infiltrating lymphocytes (TIL) populations to induce tumor rejection (20). LAG3 was almost exclusively expressed by CD8^+^ T cells. HAVCR2 was highly expressed by both suppressive tumor Tregs and exhausted CD8^+^ T cells (Figure 6F).

Our data show that Tregs highly expressed immunotherapy-related genes and that Tregs from left-sided CRC showed higher expression levels than those from right-sided CRC, indicating that left-sided CRC may be more responsive to immunotherapies that function by inducing dysfunctional Tregs (Figure 6, G and H). Pathway analysis by gene set variation analysis (GSVA) was showed in Figure 6I.

Survival analysis of the The Cancer Genome Atlas (TCGA) data set from Kaplan-Meier Plotter (http://kmplot.com/) showed that KLF2, a specific marker of Treg-C1, was an unfavorable prognostic marker (Figure 6J). DUSP1, the specific marker of Treg-C2, and RANBP1, the specific marker of Treg-C4, were favorable prognostic markers (Figure 6J). The similarity network between Tregs and other cell types in our data set is shown in Figure 6K.

### The RBP4^+^NTS^+^ cancer cell subset is unique to left-sided CRC.

**Figure 7:**
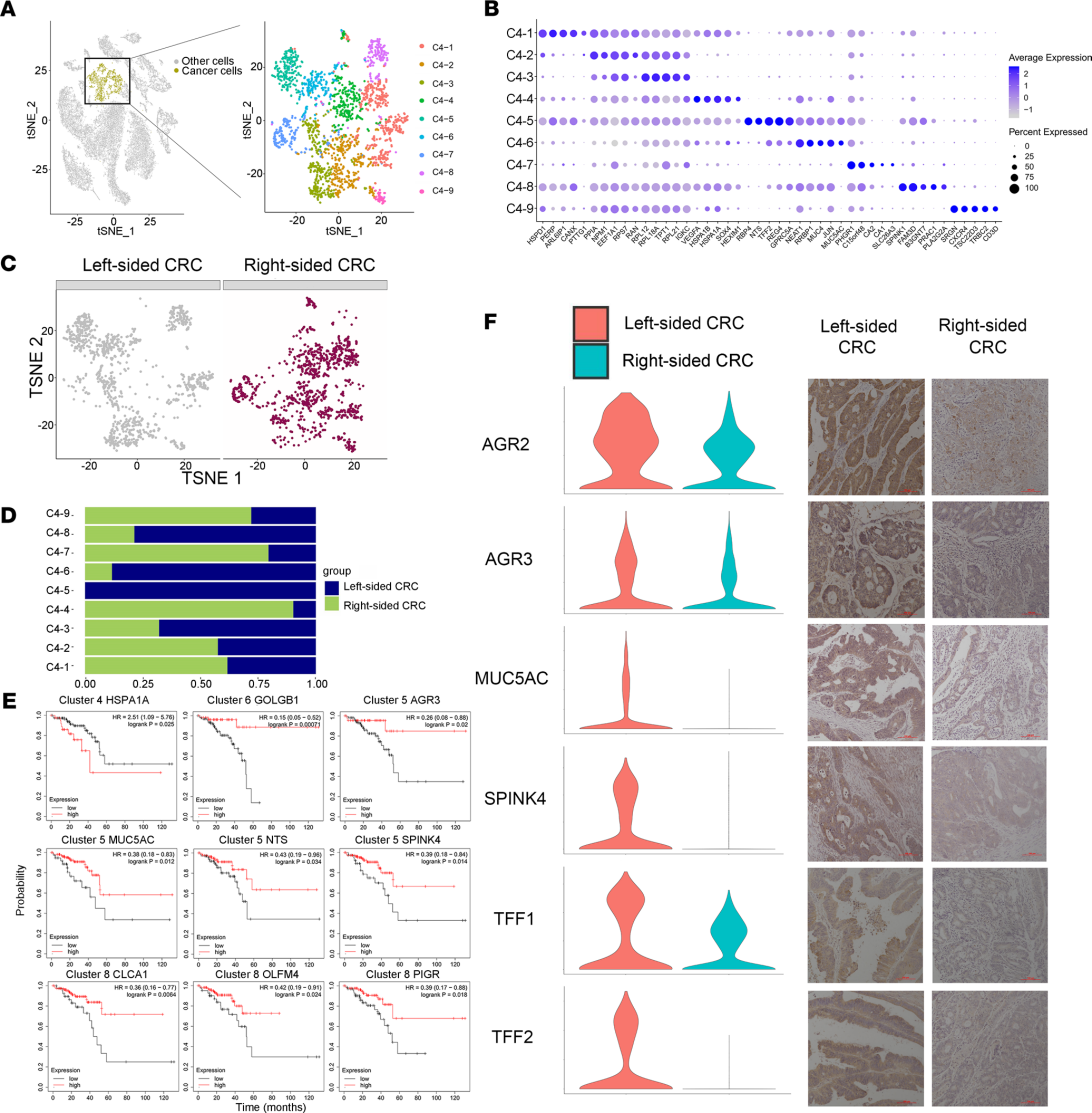
A RBP4^+^NTS^+^ cancer cell subset is unique to left-sided CRC. (**A**) The t-SNE plot that showed the distribution of cancer cell lineages (yellow, *n* = 2196 cells) within the atlas. Cancer cell populations were reclustered into 9 subclusters (color coding). (**B**) Top 5 marker genes of 15 major cell types identified in this profile. (**C**) Annotation by left-sided and right-sided CRC cells. (**D**) The fraction of cells that originated from left-sided and right-sided CRC samples for 9 subgroups identified in this profile. (**E**) Kaplan-Meier survival curves of OS based on HSPA1A, GOLGB1, AGR3, MUC5AC, NTS, SPINK4, CLCA1, OLFM4, and PIGR expression using the online bioinformatics tool Kaplan-Meier Plotter. (**F**) Violin plots and immunochemistry display the distribution of expression of AGR2, AGR3, MUC5AC, SPINK4, TFF1, and TFF2 between left-sided and right-sided CRC. Scale bars: 100 µm.

The CRC cells were further divided into 9 subgroups based on t-SNE analysis (Figure 7A). Subpopulation markers were identified across all clusters and lineages, and the top 5 markers of the main cell lineages were visualized as a bubble chart (Figure 7B). Most subgroups originated from both left-sided and right-sided CRC (Figure 7C).

Strikingly, subgroup 5 was exclusively observed in left-sided CRC patients, which reflects the high tumor heterogeneity between left-sided and right-sided CRC patients (Figure 7D). Subgroup 5 was enriched for the expression of many genes, such as RBP4, NTS, TFF2, REG4, TFF1, SPINK4, GPRC5A, AGR2, AREG, and TFF3 (top 10 specific genes in subgroup 5; Figure 7B). In addition, AGR3 and MUC5AC were also specific markers for subgroup 5 (Supplemental Figure 3). TFF1, TFF2, and MUC5AC are closely associated with protecting the mucosa from insults by stabilizing the mucus layer, thus promoting the healing process of the colorectal epithelium (21, 22).

Survival analysis of the TCGA data set showed that AGR3, MUC5AC, NTS, and SPINK4 were favorable prognostic markers (Figure 7E). Moreover, AGR2, AGR3, TFF1, TFF2, MUC5AC, and SPINK4 were expressed at higher levels in left-sided than in right-sided CRC, which was verified by IHC (Figure 7F). Survival analysis of GOLGB1, which was specifically expressed in cluster 6, was also a favorable prognostic marker. Reduced GOLGB1 expression has been reported to promote the progression of prostate cancer (23). Survival analysis of CLCA1, OLFM4, and PIGR, which were specifically expressed in cluster 8, indicated that they were favorable prognostic markers. It has been reported that increased expression of CLCA1 can suppress CRC aggressiveness (24). Survival analysis of HSPA1A, which was specifically expressed in cluster 4, indicated that it was a poor prognostic factor.

**Figure 8:**
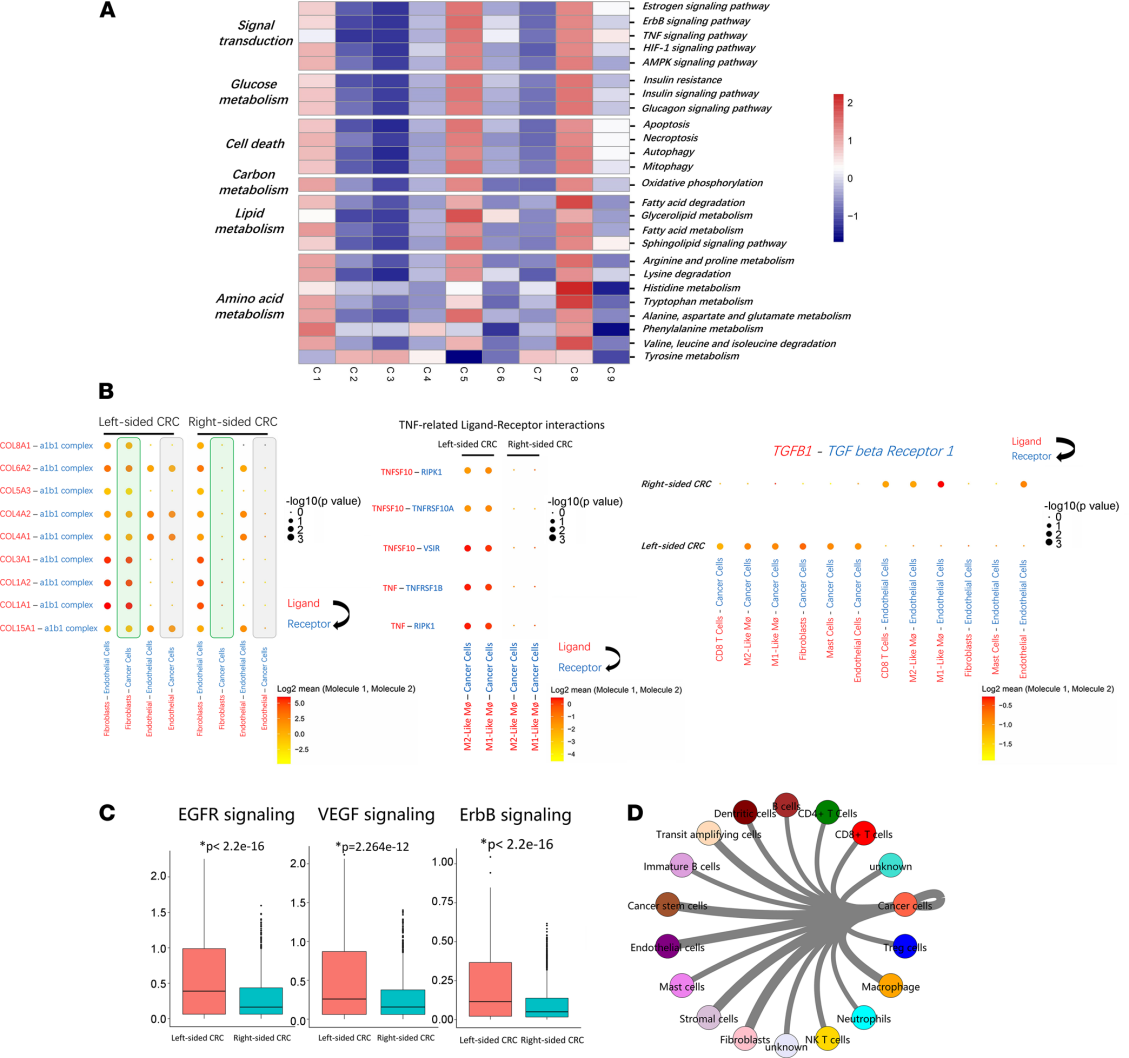
A RBP4^+^NTS^+^ cancer cell subset is unique to left-sided CRC. (**A**) Differences in hallmark pathway activities scored with GSVA software. Shown are *t* values calculated by a linear model. (**B**) Ligand-receptor interaction between cancer cells and TME-infiltrated cell clusters detected by CellPhoneDB 2. Selected ligand-receptor pairs are shown in the bubble plot. (**C**) Box plots of the expressions of Kyoto Encyclopedia of Genes and Genomes (KEGG) pathways enriched differentially expressed genes of cancer cell cluster between left-sided and right-sided CRC. (**D**) The similarity network between cancer cell and diverse cell types in our data set. The thickness of edges in the network was denoted as the Pearson correlation coefficient between the centroids of any pair of cell types. *P < 0.05. Two-tailed paired Student’s *t* test was used to determine significance.

Mapping changes in KEGG pathways during CRC revealed distinctive metabolic characteristics in 9 subgroups of cancer cells. Strikingly, cancer cell subgroup 5, which exclusively originated from left-sided CRC, presented upregulation of several cancer-associated signaling pathways, including estrogen signaling, ErbB signaling, TNF signaling, HIF-1 signaling, and AMPK signaling. The expression of estrogen receptor β has an inverse relationship with the stage of CRC and can mediate a protective response by promoting apoptosis (25). Anti-HER2 therapy may have a potentially beneficial role in the treatment of HER2^+^ metastatic CRC (26). Upregulation of ErbB signaling indicated the subgroup 5 and left-sided CRC patients may have satisfactory responses to anti-HER2 therapy. Upregulation of TNF signaling in subgroup 5 suggested a good prognosis for left-sided CRC (Figure 8A).

Furthermore, subgroup 5 showed upregulation of the cell death pathway, including apoptosis, necroptosis, autophagy, and mitophagy, indicating a good prognosis for left-sided CRC (Figure 8A). The evasion of controlled cell death induction is considered one of the hallmarks of cancer cells (27). Subgroup 5 showed upregulation of lipid metabolism, amino acid metabolism, and oxidative phosphorylation (Figure 8A).

Finally, we utilized CellPhoneDB to investigate the interactions between cancer cells and cell subgroups in the TME. As shown in Figure 8B, several collagen-encoding genes secreted by fibroblasts interact with the receptor (a1b1 complex) expressed on cancer cells, and these ligand-receptor pairs were dramatically upregulated in left-sided CRC compared with right-sided CRC. TNF and TNFSF10 (TRAIL) secreted by M1-like and M2-like macrophages interact with their receptors expressed on cancer cells. These ligand-receptor pairs associated with the TNF signaling pathway were dramatically upregulated in left-sided CRC. TRAIL is a potent anticancer agent owing to its specific targeting of cancerous cells to induce apoptosis while sparing normal cells (28). TGFβ1 secreted by the TME interacts with TGFβ receptor 1 expressed on cancer cells, and this ligand-receptor pair is dramatically upregulated in left-sided CRC. On the other hand, TGFβ1 secreted by the TME interacts with TGFβ receptor 1 expressed on endothelial cells, and this ligand-receptor pair is dramatically upregulated in right-sided CRC. TGFβ expressed in the colon plays important roles as a tumor suppressor during colorectal carcinogenesis, while TGFβ expressed on endothelial cells promotes angiogenesis in CRC (29).

The inferred developmental trajectory suggested a branched structure (Supplemental Figure 4), with cancer cell–C5 positioned at the beginning of the developmental trajectory, suggesting a possible naive state.

Our data show that left-sided CRC cancer cells exhibited significantly stronger EGFR signaling, VEGF signaling, and ErbB signaling than right-sided CRC cells (Figure 8C), which is consistent with our clinical findings that left-sided CRC is more sensitive to monoclonal antibodies against EGFR, VEGF and ErbB. The network between cancer cells and other cell types is shown in Figure 8D.

### Heterogeneity of macrophages in the TME of CRC.

**Figure 9:**
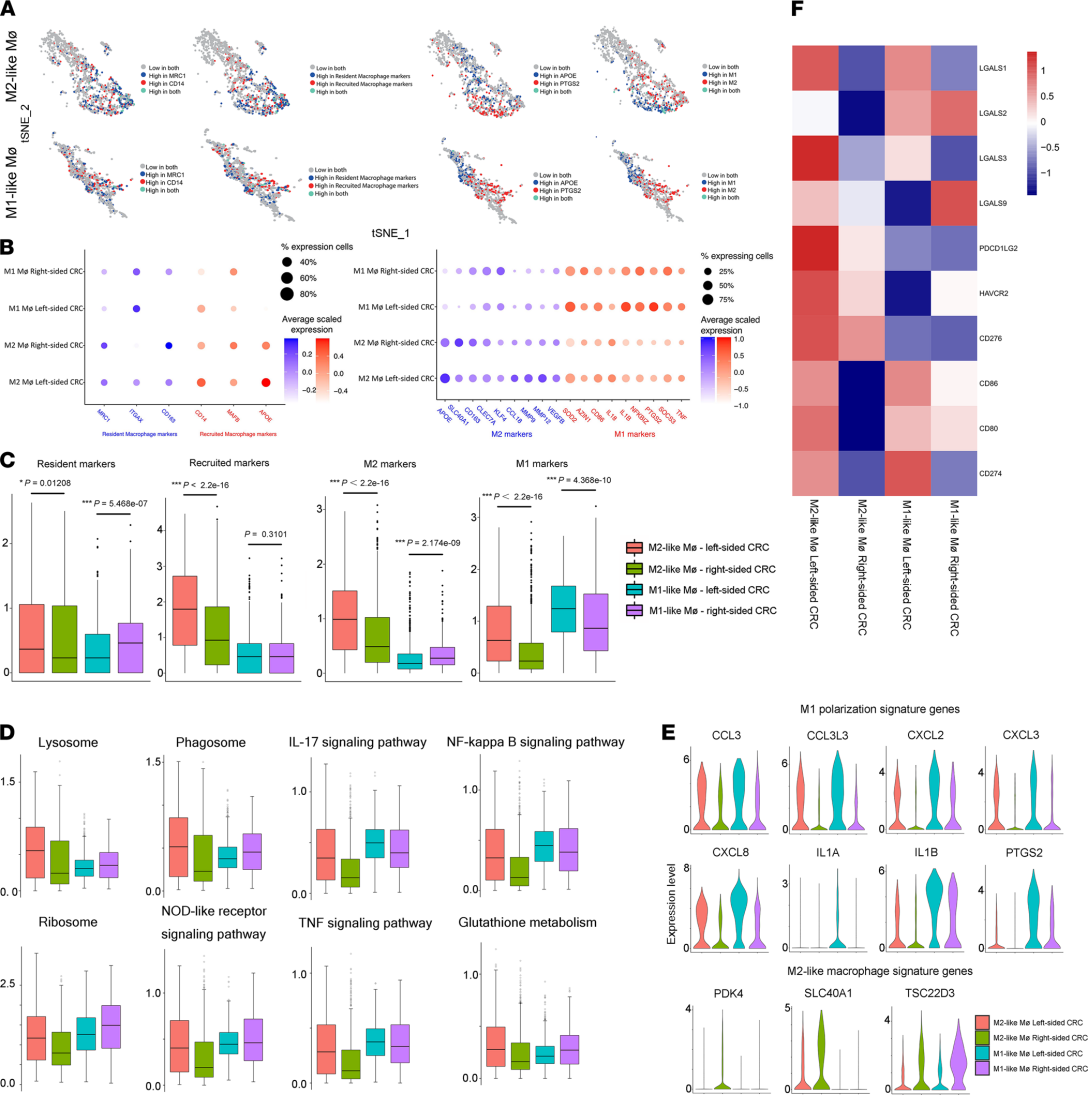
Heterogeneity of macrophages in the TME of CRC. (**A**) Relative expression of Mrc1 and CD14, APOE, and PTGS2 overlaid on t-SNE plot. Summary expression of 3 resident biomarkers (Mrc1, Itgax, and CD163) and 3 recruited biomarkers (Cd14, Apoe, and Mafb), M1 markers and M2 markers overlaid on t-SNE plot. (**B** and **C**) Bubble plot comparing expression of resident (blue) and recruited (red) biomarkers, M2 (blue) and M1 (red) markers across M1-like or M2-like macrophage clusters from left-sided or right-sided CRC. (**D**) Mean normalized expression of genes annotated for enriched pathways of M1-like and M2-like macrophage cluster from left-sided and right-sided CRC. (**E**) Violin plots display the distribution of expression of M1 and M2 polarization signature genes between left-sided and right-sided CRC. (**F**) Heatmap of positive immune checkpoint expression on macrophages. The row *Z* score was implicated to represent the expression level. *P < 0.05; **P < 0.01; ***P < 0.001. Two-tailed paired Student’s *t* test was used to determine significance.

Two transcriptionally distinct macrophage clusters were revealed. Both clusters expressed macrophage-specific markers CD68 and CD14 (Supplemental Figure 1). In virtually all tissues, resident macrophages and recruited macrophages exist during acute inflammation and carcinogenesis (30–32). We first examined the expression of MRC1 and CD14, both commonly used markers to distinguish resident macrophages and recruited macrophages. The expression of both genes was observed in both cell clusters but was greater in cluster 6, which was the M2-like macrophage (Figure 9, A–C).

We found that M1 marker genes were significantly upregulated in cluster 8 compared with cluster 6. In comparison, M2 marker genes were significantly upregulated in cluster 6 compared with clusters 8. Furthermore, the mean expression across the panel of M1 markers was 2.43-fold higher in cluster 8, whereas the mean expression of the panel of M2 markers was 3.04-fold higher in cluster 6 (Figure 9, A and B). This confirms that cluster 6 was M2-like macrophages and cluster 8 was M1-like macrophages.

Our data show that mean M1 marker expression level of M1-like macrophages from left-sided CRC was 1.43-fold higher than that of right-sided CRC, and mean M2 marker expression level of M2-like macrophages from left-sided CRC was 2.03-fold higher than that of right-sided CRC. We also noticed that M1 marker expression level of M2-like macrophages from left-sided CRC was almost the same as that of M1-like macrophages from right-sided CRC, suggesting that the M2-like macrophage group was a dynamic transitional state of M2 conversion to M1. Our data confirm that the macrophage polarization state is a major determinant of TAM heterogeneity between left-sided and right-sided CRC (Figure 9C).

Pathway analysis of DEGs from clusters 6 and 8 identified pathways known to be important for several vital functions, such as cell death (ferroptosis and necroptosis), macrophage-associated cell function (oxidative phosphorylation, ribosome, spliceosome, proteasome, lysosome, phagosome, and RNA degradation), cancer-associated signaling pathway (HIF-1, NOD-like receptor, IL-17, TNF, NF-κB, oxytocin signaling), and glutathione metabolism.

Antibody-dependent cellular phagocytosis (ADCP) represents a significant mechanism in antitumor activity mediated by activated macrophages. Macrophages can kill tumor cells extracellularly via Antibody-dependent cellular cytotoxicity (ADCC) (33). Our data show that the lysosome and phagosome pathways of M2-like macrophages from left-sided CRC were stronger than those of right-sided CRC, suggesting stronger ADCP and ADCC function and a better prognosis in left-sided CRC (Figure 9D).

The expression of IL-1A, IL-1B, CCL3, PTGS2, CXCL2/3/8, and CCL3L3 in M1-like macrophages from left-sided CRC was higher than that in M1-like macrophages from right-sided CRC. This suggested higher levels of proinflammatory cytokines and chemokines in M1-like macrophages from left-sided CRC, which further proved the stronger antitumor effect of left-sided CRC–derived M1 macrophages (Figure 9E).

The expression of PDK4, SLC40A1 and TSC22D3 in from right-sided CRC was higher than that in M2-like macrophages left-sided CRC (Figure 9E). PDK4 directly enhances cell proliferation, invasion, and chemoresistance in ovarian cancer (34). High expression of SLC40A1 is positively correlated with tumor metastasis and invasion (35). Upregulation of TSC22D3 can subvert therapy-induced anticancer immunosurveillance (36).

Macrophages express ligands for checkpoint molecules, including PD-L1 (CD274) and PD-L2 (PDCD1LG2). Macrophages contribute to the immunosuppression observed in the TME, and macrophage targeting may complement the action of checkpoint blockade inhibitors (33). As shown in Figure 9F, compared with that in other clusters, the expression of immune checkpoints in M2-like macrophages from left-sided CRC was relatively higher. Since all of these immunosuppressive ligands could suppress cytotoxic T lymphocyte function, this evidence proved that the enhanced immunosuppressive properties of macrophages in left-sided CRC and indicated the likelihood of better immunotherapy responses in left-sided CRC.

## Discussion

Although the scRNA-Seq profiles of intact gastrointestinal organs, including the esophagus, stomach, and colon, have been assessed (37–39), the profiles of CRC, particularly left-sided and right-sided CRC, have not been demonstrated. To our knowledge, this is the first study to define the difference between left-sided or right-sided CRC on the basis of a single-cell atlas. For each cancer, we identified diverse cell types and defined gene expression signatures for these cell types. We also analyzed the transcriptomic changes in some cell types across different lesions. In addition, exhausted CD8^+^ T cells, macrophages and cancer cells, and cellular characteristics related to the responsiveness of left-sided or right-sided CRC to checkpoint inhibitor therapy were analyzed in depth to identify cell type–specific markers that are potentially applicable in clinical practice.

Although immunotherapy has dramatically changed the landscape of treatment for many advanced cancers, the benefit in CRC has thus far been limited to patients with microsatellite instability–high: DNA mismatch repair–deficient (MSI-H:dMMR–deficient) tumors (40). In our study, it may provide some clues to address the issue left-sided CRC without harboring MSI-H:dMMR may also benefit from immunotherapy as a result of the high expression of checkpoint molecules on M2-like macrophages and T cells.

In summary, we constructed a single-cell transcriptome atlas of left-sided and right-sided CRC. With the atlas, we characterized the expression patterns of diverse cell types in each lesion and analyzed their changes across lesions. Of note, we identified a panel of left-sided CRC cell-specific marker genes, providing a molecular basis for precise prognosis prediction. Our findings provide multidimensional insight into the responsiveness of left-sided and right-sided malignant lesions to checkpoint inhibitor therapy, which may be helpful for predicting the effectiveness of immunotherapy for left-sided and right-sided CRC and may facilitate our understanding of CRC pathogenesis and progression.

## Methods

### Clinical sample collection and preparation.

Six patients who were pathologically diagnosed with colorectal adenocarcinoma were enrolled in this study. None of the patients had autoimmune disorders or history of prior cancer. None of the patients was treated with chemotherapy, radiation or any other antitumor medicines prior to tumor resection. Available clinical characteristics of these patients are summarized in Supplemental Table 1. Clinical samples were collected from Qilu Hospital of Shandong University. The processed gene expression data can be accessed from Gene Expression Omnibus database (accession GSE188711).

### Single-cell sequencing.

The protoplast suspension was loaded into Chromium microfluidic chips with 30 v3 chemistry and barcoded with a 10× Chromium Controller (10× Genomics). RNA from the barcoded cells was subsequently reverse transcribed, and sequencing libraries were constructed with reagents from a Chromium Single Cell 30 v3 reagent kit (10× Genomics) according to the manufacturer’s instructions. Sequencing was performed with Illumina (NovaSeq 6000) according to the manufacturer’s instructions (Illumina).

### QC.

The QC was conducted as follows. (a) Remove low-quality reads. Scan the read with a 4-base wide sliding window, cutting when the average quality per base drops below 10. (b) Remove trailing low quality or *n* bases (below quality 3). (c) Remove adapters. (d) Drop reads below the 26 bases long. (e) Discard those reads that cannot form paired.

### Generation and analysis of single-cell transcriptomes.

Raw reads were demultiplexed and mapped to the reference genome by 10× Genomics Cell Ranger pipeline (https://support.10xgenomics.com/single-cell-gene-expression/software/pipelines/latest/what-is-cell-ranger) using default parameters. All downstream single-cell analyses were performed using Cell Ranger and Seurat unless mentioned specifically.

### Cellranger.

Cellranger reanalyze takes feature-barcode matrices produced by cellranger count or cellranger aggr and reruns the dimensionality reduction, clustering, and gene expression algorithms using cellranger default parameter settings.

### Seurat.

The Seurat package was used to normalize data, dimensionality reduction, clustering, and differential expression. We used Seurat alignment method canonical correlation analysis (CCA) (41) for integrated analysis of data sets. For clustering, highly variable genes were selected, and the principal components based on those genes were used to build a graph, which was segmented with a resolution of 0.6.

### Enrichment analysis of marker genes.

GO enrichment analysis of marker genes was implemented by the clusterProfiler R package. We used clusterProfiler R package to test the statistical enrichment of marker genes in KEGG pathways.

### Definition of exhaustion, naiveness, and cytotoxicity scores.

For exhaustion scores, we first used 90 well-defined T cell exhaustion markers to define the exhaustion score for CD4^+^ T cells, CD8^+^ T cells, and Tregs after *Z* score transformation. Similarly, we used the average expression (after *Z* score transformation) of 4 well-defined naive markers (CCR7, TCF7, LEF1, and SELL) and 12 cytotoxicity associated genes (PRF1, IFNG, GNLY, NKG7, GZMB, GZMA, GZMH, KLRK1, KLRB1, KLRD1, CTSW, CST7) to define the naiveness score and cytotoxicity score for both CD8^+^ and CD4^+^ T cells, respectively. After delineating the exhaustion, naiveness and cytotoxicity scores of each T cell along the trajectory, we used locally weighted scatterplot smoothing (LOESS) regression to fit the relationships between these scores with Monocle components.

### Developmental trajectory inference.

We applied the Monocle (version 2) algorithm with the genes of cell clusters as input to determine the potential lineage differentiation between diverse cell populations. The Monocle function relative2abs was used to convert transcripts per million (TPM) measurement into mRNA cell counts per cell values, and then a CellDataSet object was created with the parameter “expressionFamily = negbinomial”. Then the cell differentiation trajectory was inferred with the default parameters of Monocle after dimension reduction and cell ordering.

### IHC.

For IHC staining, sections were routinely dewaxed and hydrated; they were then treated with 3% H_2_O_2_ for 10 minutes to block endogenous peroxidase activities, and this was followed by an overnight incubation at 4°C with indicated antibodies. Slides were then washed in PBS twice and incubated with goat anti–rabbit/mouse horseradish peroxidase–conjugated secondary antibodies (GK600505, GeneTech) for 30 minutes at room temperature. Finally, slides were washed and incubated with 3,3′-diami-nobenzidine and counter stained with hematoxylin. The slides were analyzed separately by 2 pathologists without knowing the patients’ clinical information. Anti-AGR2 antibody (ab209224) and anti-SPINK4 antibody (ab121257) were purchased from Abcam. Anti-AGR3 antibody (PA5-27222) and anti-MUC5AC antibody (PA5-79705) were purchased from Thermo Fisher Scientific. Anti-TFF1 antibody (13734-1-AP) and anti-TFF2 antibody (13681-1-AP) were purchased from Proteintech.

### Pathway analysis.

The R package limma was used to identify DEGs with normalized read counts as input and donor as an additional covariate. Genes with Benjamini-Hochberg–adjusted *P* < 0.01, and the absolute log_2_ fold change (log_2_ FC) between 2 groups larger than 1 were used for DAVID (https://david.ncifcrf.gov/) pathway enrichment analysis. In addition, preranked gene set enrichment analysis (GSEA) was also performed, using a Python implementation (package gseapy), which was also used for GSEA.

### Cell-to-cell communication analysis.

CellPhoneDB is a Python-based computational analysis tool; it enables the analysis of cell-to-cell communication at the molecular level. A website version was also provided for the analysis of relatively small data sets (https://www.cellphonedb.org/). In order to investigate the molecular interaction networks among the cell types or cell clusters, CellPhoneDB was used to analyze major cell types and cell subclusters. Ligand-receptor pairs with *P* > 0.05, as determined by CellPhoneDB, were filtered, while the others were retained for evaluating the relationship between the different cell clusters.

### Availability of data and materials.

The processed gene expression data can be accessed from Gene Expression Omnibus database (accession GSE188711).

### Statistics.

Comparison of 2 groups was performed using 2-tailed paired Student’s *t* test. Statistical analyses were performed using GraphPad Prism 5.0 (GraphPad Software Inc.). A *P* value of less than 0.05 was considered significant.

### Study approval.

The use of human colorectal cancer tissues was approved by Institutional Ethics Committee in Qilu hospital of Shandong University (KYLL-202011-209-01). The study was conducted according to the principles expressed in the Declaration of Helsinki. All patients in this study provided written informed consent for sample collection and data analyses. 

## Author contributions

WG, CZ, XW, DD, and DC performed the experiments; WG and CZ analyzed data; WG provided the samples; WG wrote the paper; JL commented on the study and revised the paper; and WG and JL designed the research.

## Supplementary Material

Supplemental data

## Version 1 11/18/2021

In-Press Preview

## Version 2 01/11/2022

Electronic publication
