# Supplementary material for: Resolving the difference between left-sided and right-sided colorectal cancer by single-cell sequencing
Source: JCI Insight. 2022 Jan 11;7(1):e152616. doi: 10.1172/jci.insight.152616 (PMC8765049; doi:10.1172/jci.insight.152616)
Supplement: Supplemental data [file jciinsight-7-152616-s087.pdf]

Figure S1

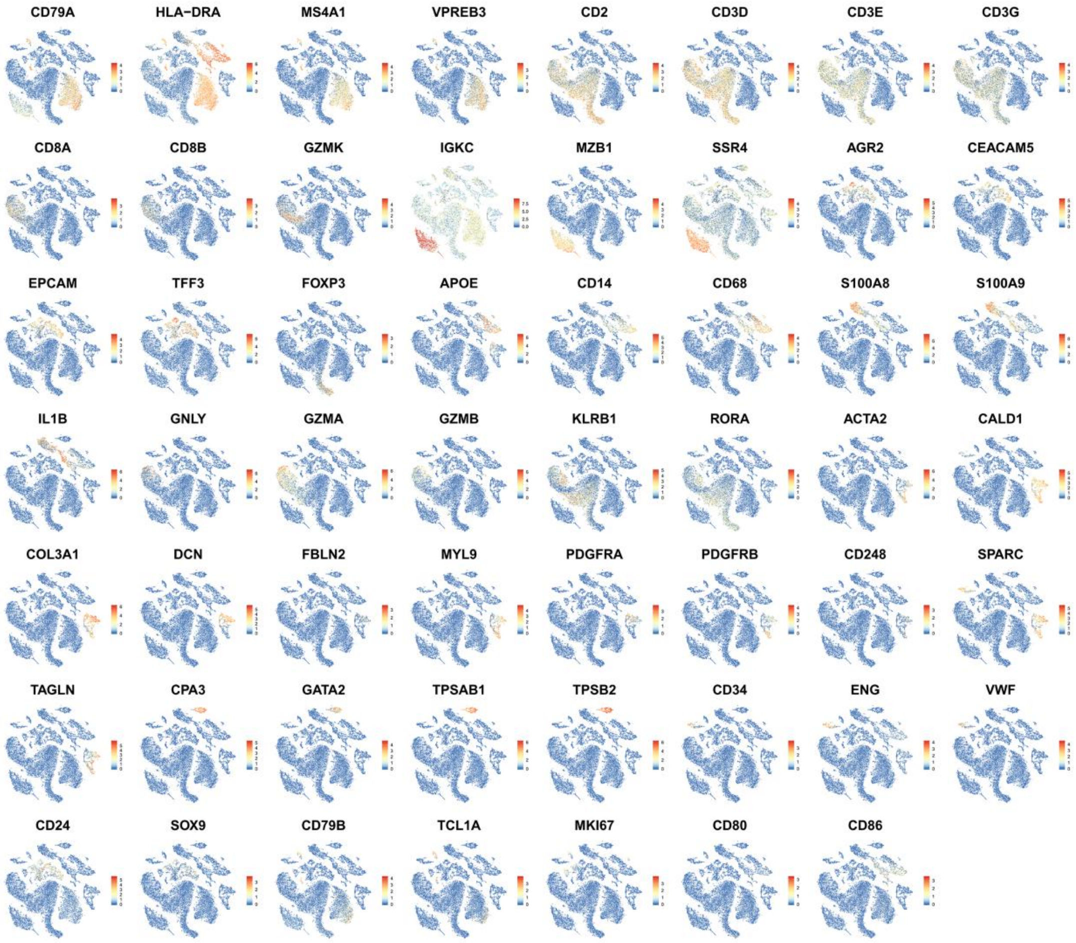

Figure S1 Selected gene expression in 27,927 T cells.

Figure S2

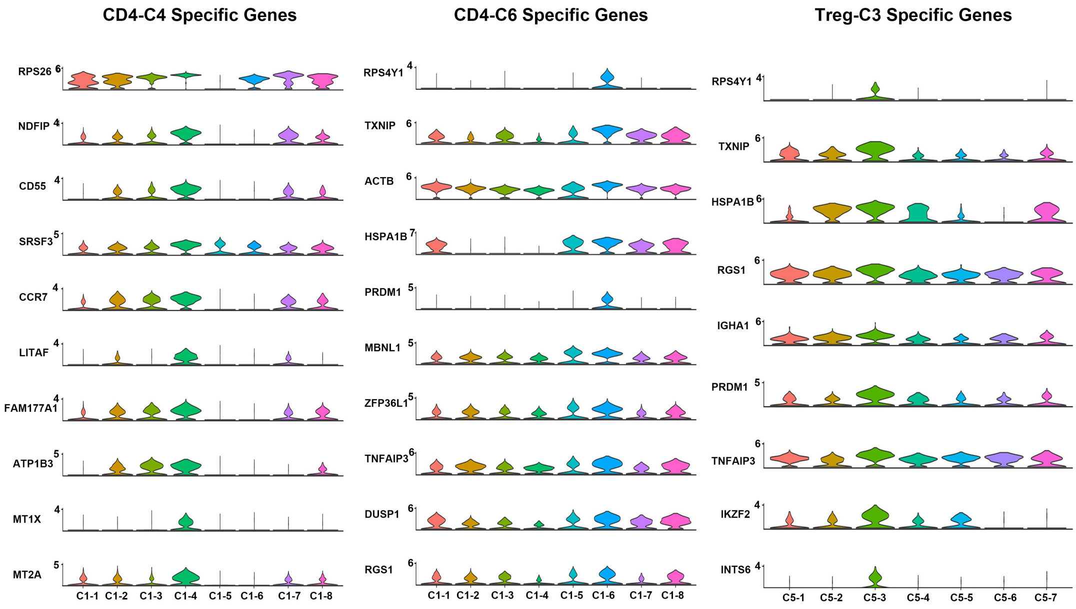

Figure S2 Violin plots of specific genes of CD4-C4, CD4-C6 and Treg-C3.

Figure S3

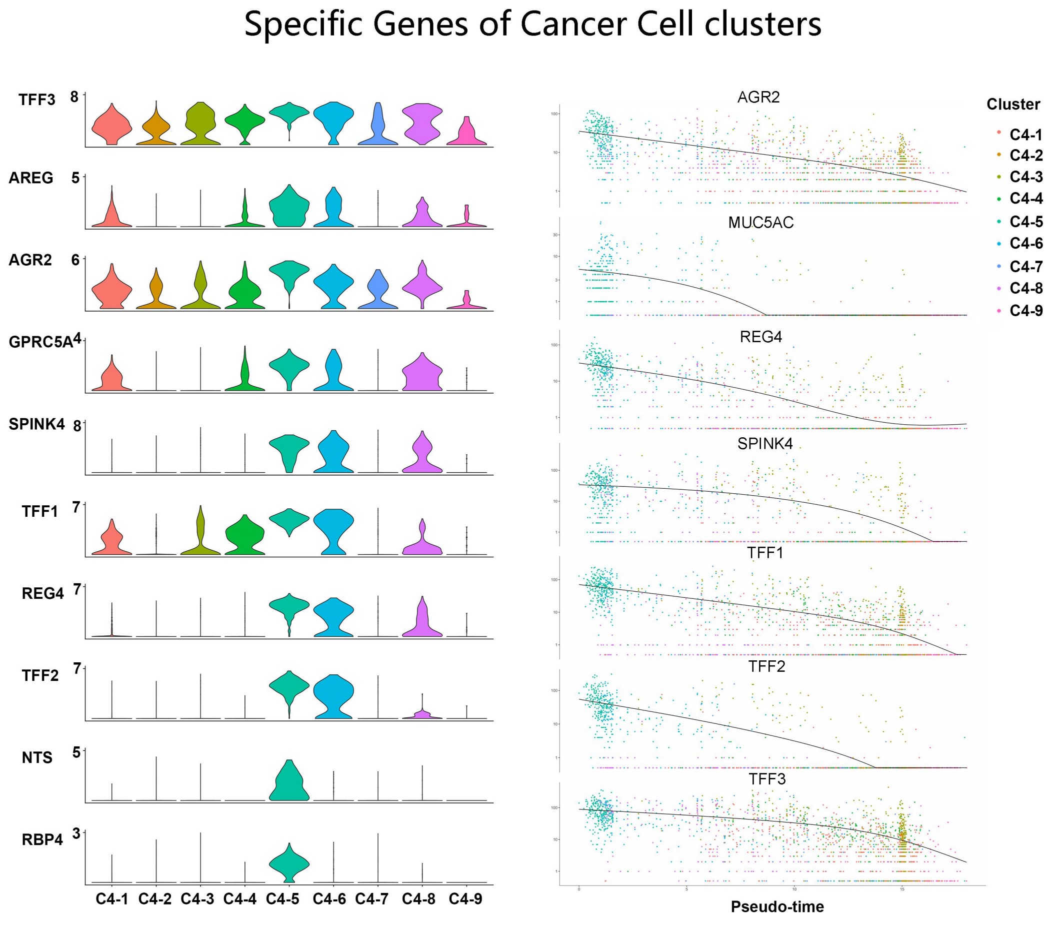

Figure S3 Specific genes of cancer cell cluster.

Figure S4

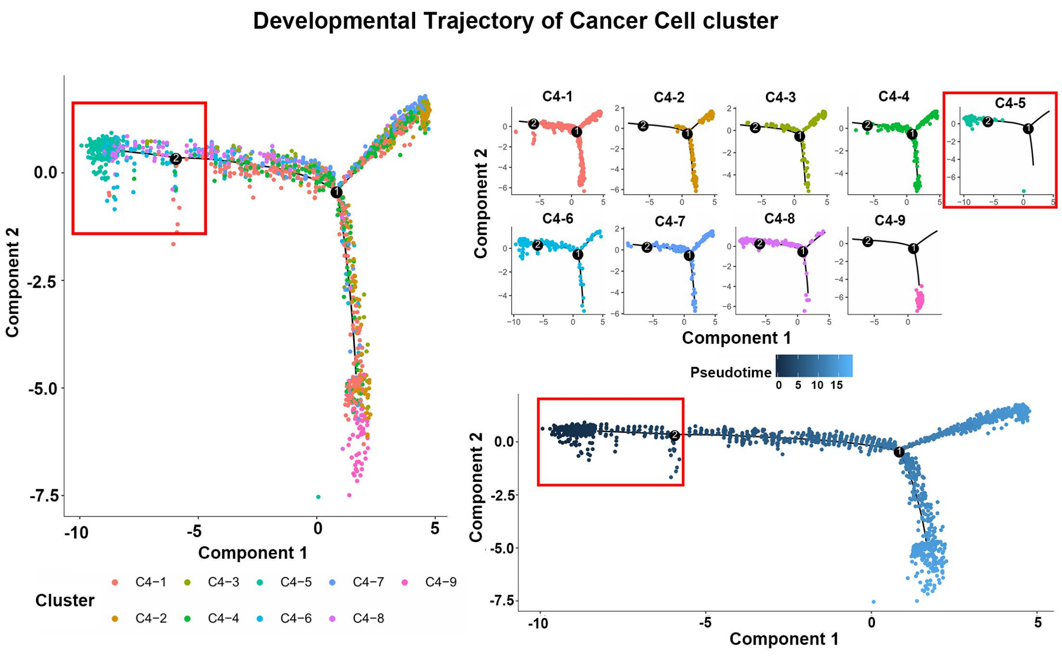

Figure S4 Developmental trajectory for cancer cell cluster.
